# Baseline MRI-based radiomics model assisted predicting disease progression in nasopharyngeal carcinoma patients with complete response after treatment

**DOI:** 10.1186/s40644-022-00448-4

**Published:** 2022-01-28

**Authors:** Dan Bao, Zhou Liu, Yayuan Geng, Lin Li, Haijun Xu, Ya Zhang, Lei Hu, Xinming Zhao, Yanfeng Zhao, Dehong Luo

**Affiliations:** 1 Department of Radiology, National Cancer Center/National Clinical Research Center for Cancer/Cancer Hospital, Chinese Academy of Medical Sciences and Peking Union Medical College, Beijing, 100021 China; 2 Department of Radiology, National Cancer Center/National Clinical Research Center for Cancer/Cancer Hospital & Shenzhen Hospital, Chinese Academy of Medical Sciences and Peking Union Medical College, Shenzhen, 518116 China; 3 Huiying Medical Technology (Beijing) Co., Ltd, B-2 Building, Dongsheng Science Park, HaiDian District, Beijing, 100192 People’s Republic of China

**Keywords:** Magnetic resonance imaging, Radiomic, Nasopharyngeal carcinoma, Disease progression, Logistic regression analysis

## Abstract

**Background:**

Accurate pretreatment prediction for disease progression of nasopharyngeal carcinoma is key to intensify therapeutic strategies to high-risk individuals. Our aim was to evaluate the value of baseline MRI-based radiomics machine-learning models in predicting the disease progression in nasopharyngeal carcinoma patients who achieved complete response after treatment.

**Methods:**

In this retrospective study, 171 patients with pathologically confirmed nasopharyngeal carcinoma were included. Using hold-out cross validation scheme (7:3), relevant radiomic features were selected with the least absolute shrinkage and selection operator method based on baseline T2-weighted fat suppression and contrast-enhanced T1-weighted images in the training cohort. After Pearson’s correlation analysis of selected radiomic features, multivariate logistic regression analysis was applied to radiomic features and clinical characteristics selection. Logistic regression analysis and support vector machine classifier were utilized to build the predictive model respectively. The predictive accuracy of the model was evaluated by ROC analysis along with sensitivity, specificity and AUC calculated in the validation cohort.

**Results:**

A prediction model using logistic regression analysis comprising 4 radiomics features (HGLZE_T2H, HGLZE_T1, LDLGLE_T1, and GLNU_T1) and 5 clinical features (histology, T stage, N stage, smoking history, and age) showed the best performance with an AUC of 0.75 in the training cohort (95% CI: 0.66–0.83) and 0.77 in the validation cohort (95% CI: 0.64–0.90). The nine independent impact factors were entered into the nomogram. The calibration curves for probability of 3-year disease progression showed good agreement. The features of this prediction model showed satisfactory clinical utility with decision curve analysis.

**Conclusions:**

A radiomics model derived from pretreatment MR showed good performance for predicting disease progression in nasopharyngeal carcinoma and may help to improve clinical decision making.

**Supplementary Information:**

The online version contains supplementary material available at 10.1186/s40644-022-00448-4.

## Background

Nasopharyngeal carcinoma (NPC), the most common tumor in the nasopharynx, prevails in east and southeast Asia, with an incidence of up to an age-standardized rate of 3.0 per 100,000 in China [1]. Intensity modulated radiotherapy is now the recommended standard treatment for non-metastatic NPC [2]. Despite proven effectiveness of radiotherapy and chemotherapy, treatment failure due to locoregional recurrence or distant metastasis occurs in nearly 10–15% of patients during the first 2 years after tumor remission [3]. It is vital to identify those NPC patients with high risk of disease progression after remission, so as to individualize treatment plan and thus better manage NPC.

Tumor-node-metastasis (TNM) staging system has been widely used to predict prognosis of patients with NPC. However, NPCs with same TNM stage can have completely different responses to chemoradiotherapy and prognosis [4], part of which may be attributed to the fact that TNM staging system mainly reflects the relationship between tumor and surrounding anatomical structures and ignores intra-tumor characteristics, including tumor morphology itself, morphological heterogeneity, etc. [5].

Conventional radiological imaging modalities, such as CT, MRI and PET-CT are widely used in the evaluation of primary tumor extension and tumor staging of NPC. These multi-modality images contain more than just anatomical information about the primary NPC lesion and its surrounding structures, and they also can reflect intra-tumor characteristics, including morphological heterogeneity that is difficult to be quantified using conventionally visual-based reading strategy [6]. As a result, without sufficient data mining, the prognosis value of these medical images was not fully utilized. Radiomics, as an emerging field of medical imaging analysis, provides a brand-new way to non-invasively quantify high-dimensional imaging features that used to be nearly impossible to be quantified by our bare eyes, and thus could provide valuable evidence to improve decision-making in cancer management [7]. Multiple studies have demonstrated the robustness of developing a radiomic signature as a prognostic tool for various cancers [8, 9], including NPC [10]. However, these early reports either mainly focused on advanced NPC [11], or only predicted local-regional recurrence without metastasis in some studies [12, 13], or just focused on distant metastasis [5]. The same as radiomics, deep learning (DL) has also become one of the most important artificial intelligence (AI) tools, whose application in NPC have been gradually increasing since 2017 [14]. Jing [15] established an end-to-end multi-modality deep survival network (MDSN) to predict the risk of disease progression of NPC patients, but the best performance was a C-index of 0.651. A deep convolution model based on ResNet was established to predict the distant metastasis-free survival of locoregionally advanced NPC patients [16]. In this study, there was no significant difference between deep learning and radiomic signatures when they were used independently. The optimal AUC of the multiple models combined with the clinical features was 0.808. Therefore, it is necessary to establish and evaluate more comprehensive radiomics models based on baseline MRI of NPC in different clinical stages to predict the risk of disease progression of NPC to guide individualized treatment.

Herein, the purpose of this study was to evaluate the value of multiple radiomics models based on multi-parametric MR images at baseline in predicting disease progression probability of non-metastatic NPC after tumor remission. We present the following article in accordance with the STARD reporting checklist.

## Methods

### Patient selection and clinical characteristics

**Fig. 1:**
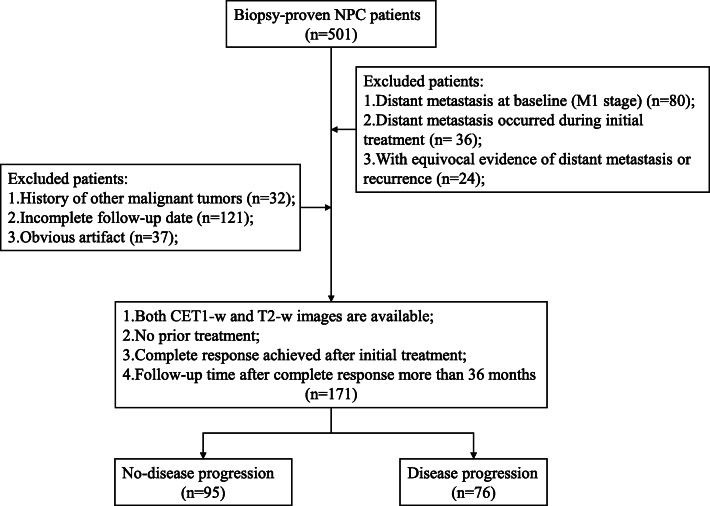
Flow-chart for patient inclusion

This retrospective study was approved by the institutional review board and the requirement for written informed consent was waived. Between January 2012 and July 2016, a total of 501 patients with NPC confirmed by pathological examinations were identified from the Picture Archiving and Communication System (PACS) of our institution. We only included those patients: 1) who underwent nasopharynx-neck MRI within 2 weeks before any type of antitumor treatment; 2) with no apparent artifacts of any type on MR images that may affect imaging analysis; 3) without evidence suggesting distant metastasis in the baseline assessment; 4) who achieved a complete response after initial treatment; 5) who were then followed up for at least 36 months after complete response. A total of 171 consecutive NPC patients who met the inclusion criteria were included in this study (Fig. 1). During the 36 months after complete response to treatment, NPC patients who developed local-regional recurrence or distant metastasis were assigned to disease progression group, otherwise to non-disease progression group.

From the medical records, clinical characteristics were collected including age, gender, histopathology, T-stage, N-stage, overall clinical stage, treatment regimen, pretreatment anti-EBV immunoglobulin A antibody (including early antigen immunoglobulin A [EA-IgA] and anti-viral capsid antigen immunoglobulin A [VCA-IgA]) and regular smoking history. Two senior radiologists (D.L. and Y.Z., with 31 and 17 years of diagnostic radiology experience respectively) performed tumor staging based on nasopharyngoscopy result and medical images according to the TNM classification (7th edition American Joint Committee on Cancer/Union for International Cancer Control) [17], with any disagreements resolved through discussion.

### Treatment regimen and follow-up

All patients underwent an individualized treatment based on NCCN guideline [18]. The total radiation doses ranged from 66 to 75 Gy (mean, 70 Gy). All patients received treatment covering the primary tumor, cervical adenopathy, and adjacent at-risk tissues. Patients with stage I tumors received radiotherapy alone, while stage II to IVA patients usually were recommended to have neoadjuvant or adjuvant chemotherapy and/or concurrent chemotherapy with radiotherapy.

Three months after the completion of treatment, treatment response was evaluated for each patient with complete response indicated by MRI and confirmed by nasopharyngoscopy with biopsy. Patients with complete response after the completion of treatment were then followed up at least every 3 months during the first 2 years and every 6 months starting from the third year ever since the initiation of treatment. The routine clinical follow-up work-ups including physical examination, nasopharyngoscopy, one or multiple of imaging modalities, such as CT, MRI, PET/CT and whole-body bone scintigraphy were performed for each patient. Since over 90% of metastases occurred in the first 3 years after the initiation of treatment for NPCs, the cut-off follow-up time was arbitrarily set as 36 months, during which disease progression was defined as the primary clinical endpoint. Disease progression included local-regional recurrence and distant metastasis. Local recurrence for NPC was defined as recurrence at the primary site following complete response after treatment completion, which was confirmed by biopsy and/or MRI of the nasopharynx [12]. Regional recurrences were confirmed using fine needle aspiration biopsy when clinical examination of the neck and MRI indicated progressive cervical adenopathy. Distant metastases were diagnosed based on clinical symptoms, physical examination and imaging findings on nasopharyngeal-neck MRI, thoracic CT, abdominal sonography and whole-body bone scintigraphy, PET-CT, as appropriate.

### MRI scan acquisition and tumor segmentation

All of the MR images were acquired using 3.0 T MR scanners (GE Discovery MR 750, General Electric Medical Systems, US) with an 8-channel head and neck phase array coil. For feature extraction, we used axial fast spin echo (FSE) T2-weighted with fat saturation (T2/FS-w) Digital Imaging and Communications in Medicine (DICOM) images and axial fast spoiled gradient-echo (FSPGR) contrast-enhanced T1-weighted (CET1-w) DICOM images that had been archived using PACS. Axial FSPGR CE-T1w imaging was obtained 50–60s after intravenous bolus injection of gadopentetate dimeglumine (Magnevist, Bayer, Leverkusen, Germany) at a dosage of 0.2 ml/kg of body weight at 2.5 ml/second with a power injector. The acquisition parameters were as follows: axial T2-weighted spin-echo images (repetition time [TR]/echo time [TE]: 8472/85 ms, field of view [FOV] = 24 × 24 cm, number of excitations [NEX] = 2.0, slice thickness = 4 mm, spacing between slices = 1.0 mm) and axial contrast-enhanced T1-weighted gradient-echo images (TR/TE: 315/2.9 ms, FOV = 24 × 24 cm, NEX = 1.0, slice thickness = 4 mm, spacing between slices = 1.0 mm).

**Fig. 2:**
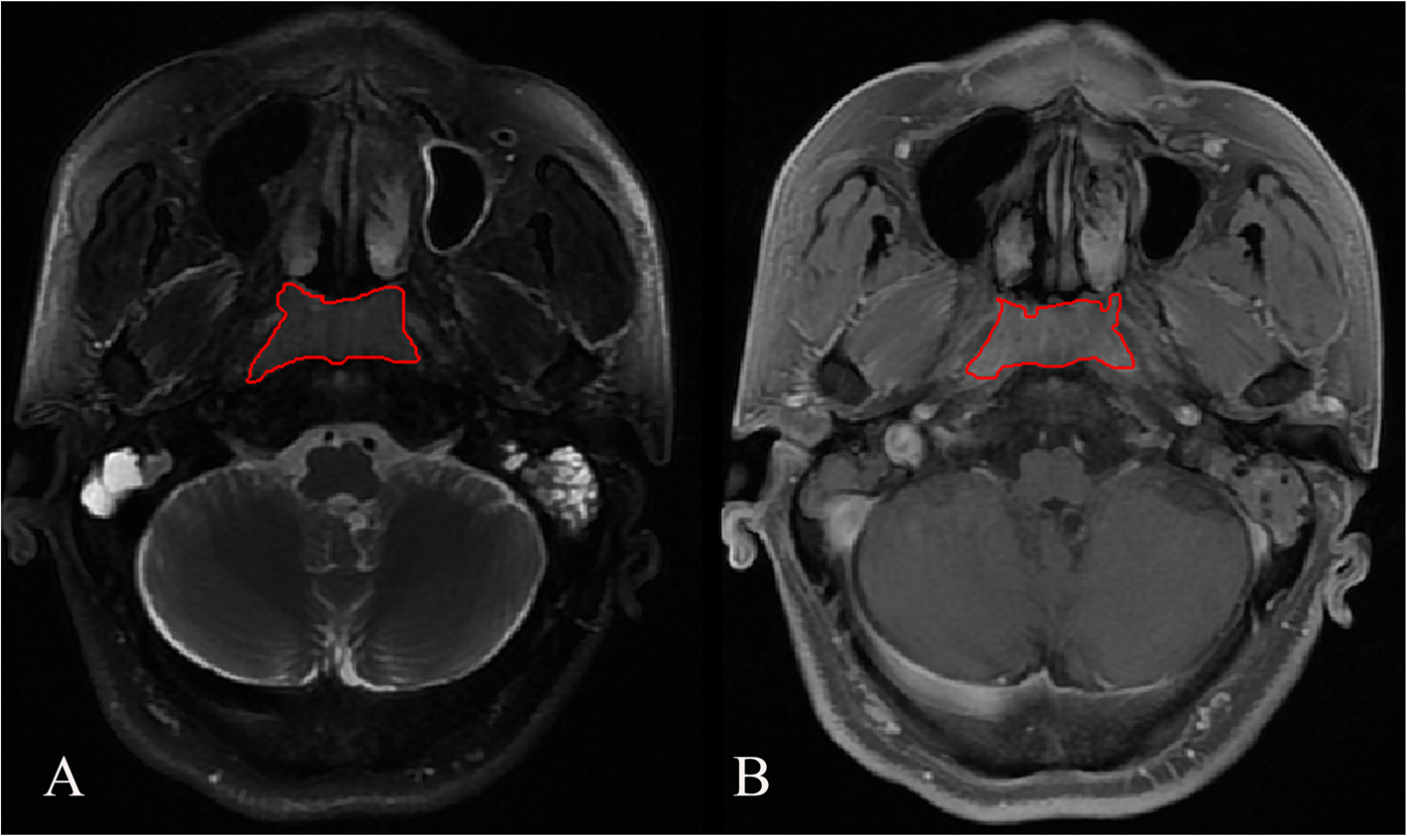
An example of manual segmentation in a 56-year-old male patient with NPC. The segmented tumor is within the red contour in one slice of oblique axial T2WI/FS sequence (**A**) and the red contour in one slice of axial CE-T1WI sequence (**B**)

The VOIs were based on the primary NPC lesions on the T2/FS-w and CET1-w images separately. Tumor segmentation was manually performed by a radiologist (D.B. with 3 years of experience in head and neck imaging) on the radiomics cloud platform V2.1.2 (Huiying Medical Technology Co., Ltd.), which were then reviewed by a senior radiologist (D.L.) who is specialized in head and neck imaging with 31 years of experience. For each lesion, the VOIs were drawn to cover the whole NPC tumor on each consecutive slice, with necrosis, hemorrhage, and cystic areas excluded (Fig. 2; Additional Fig. 1). The interobserver variability in manual segmentation between different readers was assessed. Two radiologists (D.B. and L.H.; with 4 and 6 years of experience, respectively) independently manually segmented the VOIs from the MR images of 30 patients randomly selected from the whole sample at a ratio of one-to-one between the two groups. The Dice similarity coefficient (DSC) for absolute agreement was calculated between segmented VOIs from each patient. According to the guidelines, Dice index of < 0.6, 0.6–0.8, 0.8–1.0, and 1.0 indicates inadequate, good, very good, and ideal consistency, respectively [19]. Then, intraclass correlation coefficient (ICC) for absolute agreement was calculated between radiomics features extracted from the VOIs of 30 randomly selected cases. An ICC above 0.75 was indicative of good agreement [20].

### Features extraction and dimensionality reduction

On the radiomics cloud platform V2.1.2 (Huiying Medical Technology Co., Ltd., the Python software, version 3.7.1) a total of 1409 features classified into four different feature classes were extracted, including intensity statistics features, shape- and size-based features, textural features, and filter and wavelet features. To ensure spatial consistency in texture analysis across the images, all images normalized by centering to the mean standard deviation, resampled to voxel size of 1 × 1 × 1 mm^3^ using B-Spline interpolation, and gray-level discretized by a fixed bin width of 25 in the histogram. Most feature extraction methods conform to the Imaging Biomarker Standardization Initiative (IBSI) standard [21]. With hold-out cross validation scheme (training set: validation set = 7:3, the number of seed = 1234) being used, the least absolute shrinkage and selection operator (LASSO) regression algorithm was used to reduce irrelevant features in the training set.

### Feature selection methodology

To further reduce the redundancy of radiomic features in the training set, we performed Pearson correlation analysis with one of the paired features having correlation coefficient of more than 0.9 removed [22]. The remaining radiomic features and all clinical variables form a stable and reliable primary dataset. Then, the multivariate logistic regression analysis was performed for feature selection from the dataset with *P* < 0.05 indicating independent significant variable. Radiomics and clinical features that were independently significantly associated with disease progression were selected for the final classification modeling on the training set.

### Construction and validation of radiomics-based model

**Fig. 3:**
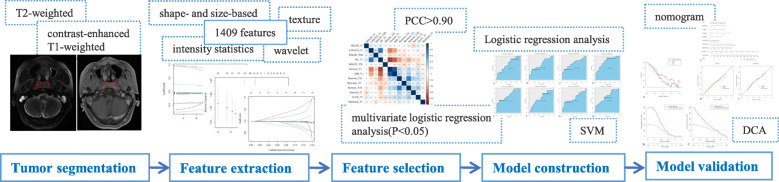
Workflow of the radiomic analysis in the current study

Logistic regression analysis and support vector machine (SVM) classifier were applied for model building respectively. On the basis of the feature selection results, we constructed a classification model called a prediction of disease progression MRI-based (PDPM) model (in R with the glm package). An overview of the radiomics analysis process is presented in Fig. 3.

We drafted a nomogram integrating the variables included in the best PDPM model, in R with the rms package, which could visualize the weight of different variables in combined model. The area under the receiver operating characteristic curve (AUC) and its 95% confidence interval (CI) was calculated, which tested the model’s ability to discriminate patients with or without disease progression [23]. Nomogram was applied to reduce the statistical predictive model into a single numerical estimate of the probability of disease progression [24]. Calibration curves, which indicated the calibration ability of the nomogram, were assessed graphically by plotting the actual observed survival rates and the nomogram-predicted survival rates via a bootstrap method with 1000-iteration resampling.

In addition, decision curves analysis (DCA), in R with the rmda package, was introduced to estimate clinical utility of PDPM models based on the threshold probability (probability that triggers a medical intervention by a clinician or patient, equating to the probability at which the harm of a false-positive intervention exceeds the harm of a false-negative non-intervention) [25].

### Statistical analysis

The Shapiro-Wilk test was used to determine whether the distribution of all the variables was normal, and Levene’s test was used for identifying the homogeneity of variance. The clinical characteristics of the disease progression group and non-disease progression group were compared using an independent samples t-test or the Mann–Whitney U test, Fisher’s exact test or the chi-squared (χ^2^) test, as appropriate. Diagnostic performance of the models was assessed by using receiver operating characteristic (ROC) (in R with the pROC package) curve quantified by the AUC, sensitivity, and specificity. The AUCs were compared across models by using the DeLong’s test. The Dice similarity coefficient (DSC) was used to test the consistency between the two radiologists on the manually segmented VOIs of 30 randomly selected samples.

The formula for Dice is as follows:

$$ Dice\left(A,B\right)=2\times \frac{\left|A\cap B\right|}{\left|A\right|+\left|B\right|} $$

∣*A*∣ and ∣*B*∣ represents the segmented VOIs based on the same NPC lesion by two radiologists, respectively. ∣*A* ∩ *B*∣ indicates intersection between the manually segmented VOI A and VOI B.

All statistical analyses were performed using SPSS software (version 21.0; IBM Corp., Armonk, NY) and R software (version 4.0.2). *P* < 0.05 was considered to indicate a statistically significant difference.

## Results

### Clinical characteristics

A total of 171 patients with pathologically confirmed NPC was included in our study. Baseline clinicopathologic characteristics are summarized in Table 1. The results of correlation analysis showed that age (*P* = 0.04), gender (*P* = 0.01) and smoking history (*P* = 0.02) were significantly associated with disease progression but not for other clinical variables (*P* > 0.05). During 3 years of follow-up, 76 patients who had developed local-regional recurrence or distant metastasis were assigned to disease progression group with the median follow-up duration of 14.2 months (range, 4–35.5 months), while 95 patients who had no local-regional recurrence or distant metastasis were assigned to non-disease progression group.

**Table 1:** Baseline clinical characteristics of the patients in the disease progression group and non-recurrence/non-disease progression group

Clinical characteristic	Disease progression group (***n*** = 76)	Non-disease progression group (***n*** = 95)	***P*** value
**Age (mean ± SD, years)**	46.0 ± 12.4	42.0 ± 12.7	0.04*
**Gender**			0.01*
Male	66	67	
Female	10	28	
**Histology**			0.07
Differentiated Non-keratinising	45	43	
Undifferentiated Non-keratinising	31	52	
**T stage**			0.79
T1	8	15	
T2	14	17	
T3	31	35	
T4	23	28	
**N stage**			0.22
N0	3	9	
N1	21	34	
N2	36	39	
N3	16	13	
**Overall stage**			0.52
I	0	1	
II	8	11	
III	28	41	
IV	40	42	
**Treatment**			0.50
A	12	10	
B	39	45	
C	3	7	
D	22	33	
**EA-IgA**			
positive	23	30	0.85
negative	53	65	
**VCA-IgA**			
positive	53	57	0.19
negative	23	38	
**Smoking**			0.02*
Yes	44	38	
No	32	57	

Treatment: A-Radiotherapy only, B-Chemotherapy + Radiotherapy, C-Targeted therapy + Radiotherapy, D-Concurrent Chemoradiotherapy + Targeted Therapy, E-Chemotherapy + Targeted Therapy; * indicates statistical significant difference

### Interobserver variability between readers

Two readers had very good agreement in the manual segmentation with Dice value of 0.85 ± 0.04 (range 0.74–0.91) for T2WI/FS sequences and 0.84 ± 0.05 (range 0.73–0.90) for CE-T1WI sequences. The inter-reader ICC between the two radiologists ranged was 0.93(range 0.83–0.97). These results indicated a favorable inter-observer reproducibility for feature extraction.

### Radiomic feature extraction and features selection

**Fig. 4:**
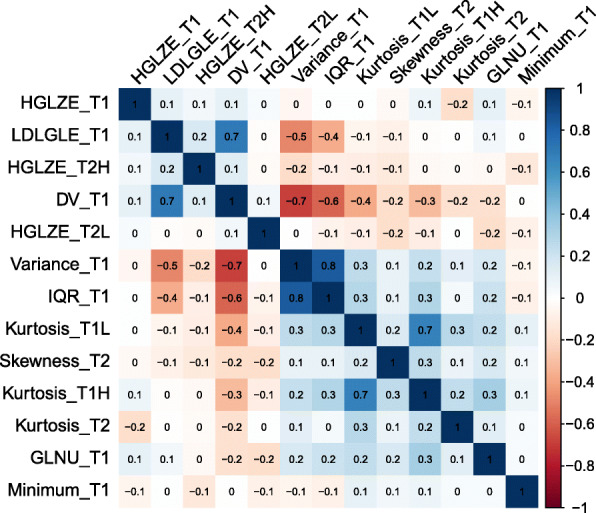
Pearson correlation coefficient of the 13 significant features

A total of 1409 radiomic features were extracted from axial T2/FS-w and CET1-w images on the training set, respectively. After the dimensionality reduction using LASSO regression algorithm, 13 radiomic features were selected. We performed Pearson correlation analysis with the 13 radiomic features to prevent overfitting. None of the 13 radiomic features was found to be significant highly correlated (all r < 0.90) (Fig. 4). A detailed description list of these 13 radiomic features is presented in Table 2. The 13 radiomic features and 10 clinical variables form a stable and reliable primary dataset. No significant differences in clinical and radiomic features of patients were observed between the training and validation cohorts, as shown in Table 3 (all *P* > 0.05). We included the primary dataset in multivariate logistic regression analysis, HGLZE_T2H (*P* = 0.03), HGLZE_T1 (*P* = 0.01), LDLGLE_T1 (P = 0.01), GLNU_T1 (P = 0.03), histology (*P* = 0.02), T stage (P = 0.03), N stage (P = 0.02), and smoking history (*P* = 0.003) were found to be significantly associated with disease progression. Based on the result of univariate analysis of clinical characteristics and clinical consensus, we also included age as one of the clinical variables in the final modeling. Finally, we constructed 4 variable sets according to the results of feature selection for subsequent PDPM model establishment, 1) all 13 radiomic features selected and 10 clinical variables (Model 1); 2) 5 clinical variables only (Model 2), determined by multivariate logistic regression analysis and clinical consensus; 3) 4 radiomic features only (Model 3), determined by multivariate logistic regression analysis; 4) 4 radiomic features and 5 clinical variables (Model 4), determined by multivariate logistic regression analysis and clinical consensus.

**Table 2:** List of 13 radiomic features parameters

Texture type	Texture parameters	Abbreviations
First order	CET1-w_local binary pattern-2D_Variance	Variance_T1
	CET1-w_local binary pattern-2D_Interquartile Range	IQR_T1
	CET1-w_wavelet-HLL_Minimum	Minimum_T1
	CET1-w_wavelet-HHL_Kurtosis	Kurtosis_T1H
	CET1-w_wavelet-LHL_Kurtosis	Kurtosis_T1L
	T2-w_wavelet-LHH_Kurtosis	Kurtosis_T2
	T2-w_squareroot_Skewness	Skewness_T2
GLSZM	CET1-w_wavelet-LHH_Gray Level Non-Uniformity	GLNU_T1
	CET1-w_wavelet-HLL_High Gray Level Zone Emphasis	HGLZE_T1
	T2-w_wavelet-HLL_High Gray Level Zone Emphasis	HGLZE_T2H
	T2-w_wavelet-LHH_High Gray Level Zone Emphasis	HGLZE_T2L
GLDM	CET1-w_wavelet-LHL_Dependence Variance	DV_T1
	CET1-w_wavelet-LHL_Large Dependence Low Gray Level Emphasis	LDLGLE_T1

**Table 3:** The clinical and radiomic characteristics of patients in the training and validation cohorts

Characteristic	Training cohort(***n*** = 119)	Validation cohort(***n*** = 52)	***P*** value
**Age (mean ± SD, years)**	43.34 ± 12.5	44.81 ± 13.1	0.49
**Gender**			0.44
Male	95	38	
Female	24	14	
**Histology**			0.79
Differentiated Non-keratinising	62	26	
Undifferentiated Non-keratinising	57	26	
**T stage**			0.13
T1	17	6	
T2	21	10	
T3	40	26	
T4	41	10	
**N stage**			0.11
N0	10	2	
N1	41	14	
N2	53	22	
N3	15	14	
**Overall stage**			0.71
I	1	0	
II	14	5	
III	50	19	
IV	54	28	
**Treatment**			0.24
A	14	8	
B	56	28	
C	5	5	
D	44	11	
**EA-IgA**			
Positive	38	15	0.82
Negative	81	37	
**VCA-IgA**			
Positive	42	33	1.00
Negative	77	19	
**Smoking**			1.00
Yes	57	25	
No	62	27	
**Variance_T1**	7.67 ± 0.71	7.64 ± 0.70	0.77
**IQR_T1**	4.34 ± 0.76	4.37 ± 0.69	0.81
**Skewness_T2**	−1.79 ± 0.70	−1.96 ± 0.72	0.16
**Kurtosis_T1H**	6.62 ± 2.29	6.71 ± 1.55	0.80
**HGLZE_T2H**	2.87 ± 0.43	2.92 ± 0.39	0.45
**HGLZE_T1**	3.03 ± 0.41	3.16 ± 0.42	0.06
**DV_T1**	22.17 ± 2.16	22.51 ± 2.10	0.34
**Kurtosis_T1L**	8.24 ± 3.82	7.69 ± 2.92	0.35
**LDLGLE_T1**	145.67 ± 11.28	147.02 ± 11.15	0.47
**Kurtosis_T2**	9.04 ± 9.04	8.18 ± 4.83	0.52
**HGLZE_T2L**	2.55 ± 0.51	2.44 ± 0.46	0.19
**Minimum_T1**	−1.84 ± 0.44	− 1.75 ± 0.42	0.22
**GLNU_T1**	5.24 ± 2.51	5.52 ± 2.40	0.49
**Prognosis**			0.64
Disease progression	51	25	
Non-disease progression	68	27	

Treatment: A-Radiotherapy, B-Chemotherapy + Radiotherapy, C-Targeted therapy + Radiotherapy, D-Concurrent Chemoradiotherapy + Targeted Therapy, E-Chemotherapy + Targeted Therapy; * indicates statistical significant difference

### Development and performance of models

**Fig. 5:**
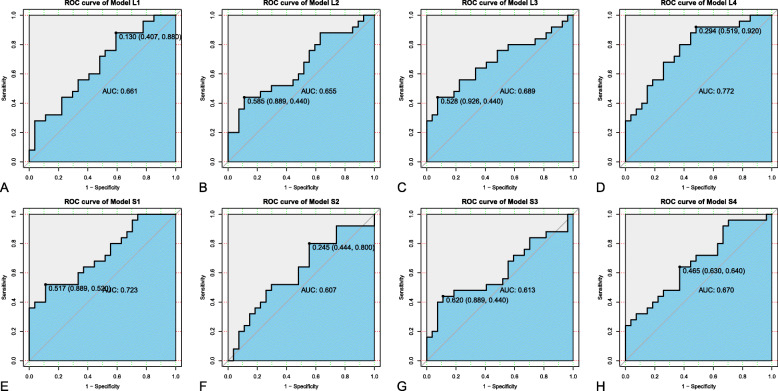
The receiver operating characteristic (ROC) curve plots of the eight prediction of disease progression MRI-based (PDPM) models in the training (**A, B, C, D**) and validation cohort (**E, F, G, H**)

Four types of PDPM models were established with logistic regression (Model L1, L2, L3, L4) and support vector machine (SVM) (Model S1, S2, S3, S4) were trained with the four variable sets to predict 3-year disease progression of patients with NPC. Prognostic performances of different PDPM models are shown in Table 4. The best performance was found in the model trained with both 4 radiomic features and 5 clinical features (Model L4), with an AUC of 0.75 in the training cohort (95% CI: 0.66–0.83) and 0.77 in the validation cohort (95% CI: 0.64–0.90). The ROC curve plot of the eight models in validation cohort are shown in Fig. 5. The Model L4 [AUC = 0.77 (95% CI: 0.64–0.90)] exhibited significantly better prediction performance than Model L1 [AUC = 0.66 (95% CI: 0.51–0.81), *P* = 0.03], Model S2 [AUC = 0.61 (95% CI: 0.45–0.76), *P* = 0.02] and Model S4 [AUC = 0.67 (95% CI: 0.52–0.82), *P* = 0.04] in the validation cohorts, respectively.

**Table 4:** Predictive performance of the PDPM models in predicting the disease progression in the cohorts

Model	Training cohort (***n*** = 119)					Validation cohort (***n*** = 52)				
	AUC	95% CI		SEN	SPE	AUC	95% CI		SEN	SPE
		Low	High				Low	High		
**L1**	0.85	0.78	0.92	0.80	0.77	0.66	0.51	0.81	0.88	0.41
**L2**	0.69	0.59	0.78	0.75	0.54	0.66	0.50	0.81	0.44	0.89
**L3**	0.66	0.57	0.76	0.96	0.32	0.69	0.54	0.84	0.44	0.93
**L4**	0.75	0.66	0.83	0.67	0.75	0.77	0.64	0.90	0.92	0.52
**S1**	0.97	0.94	1.00	0.98	0.88	0.72	0.58	0.86	0.52	0.89
**S2**	0.84	0.76	0.91	0.84	0.72	0.61	0.45	0.77	0.80	0.44
**S3**	0.85	0.78	0.92	0.71	0.75	0.61	0.45	0.77	0.44	0.89
**S4**	0.95	0.91	0.98	0.96	0.78	0.67	0.52	0.82	0.64	0.63

AUC, area under the curve; CI, confidence interval; SEN, sensitivity; SPE, specificity

### Model explanation with nomogram and DCA

**Fig. 6:**
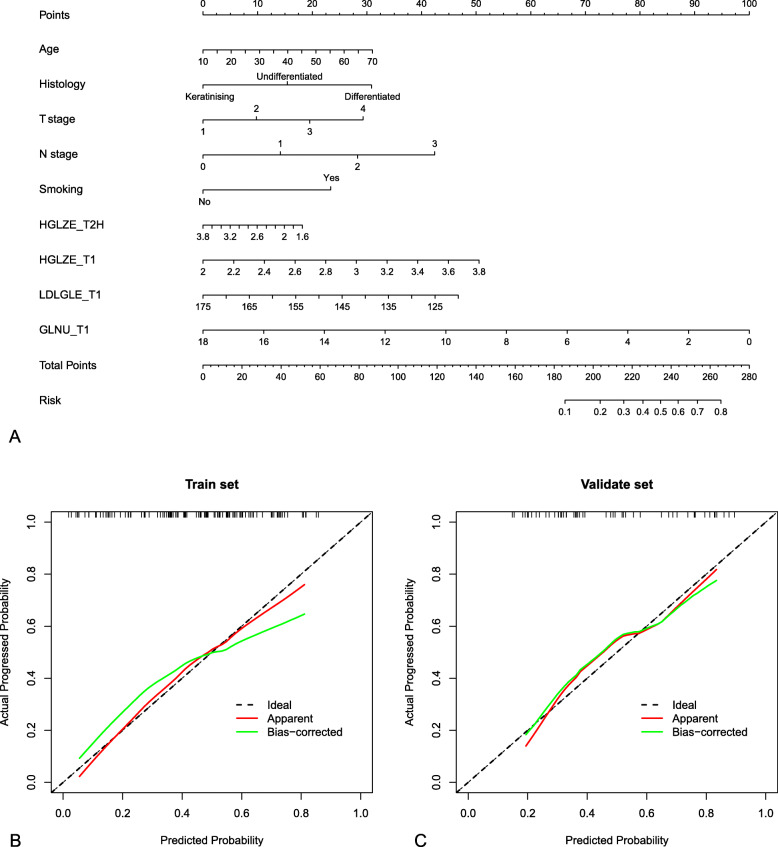
(**A**): A nomogram incorporated 4 radiomic features and 5 clinical features of Model L4. (**B, C**): Calibration curves of nomogram developed in the training and validation cohorts

To provide the clinician with a quantitative method for prediction the risk of disease progression in NPC patients, we constructed a nomogram integrating 4 radiomic features and 5 clinic features included in Model L4 (Fig. 6A). Calibration estimates how close the nomogram estimated risk is to the observed risk, depicted by a calibration plot (Fig. 6B and C). Good calibration was observed for the probability of disease progression in the training cohort and validation cohort. Calibration varies with nomogram calculated probabilities. For instance, the nomogram is more accurate at predicting a disease progression of 50% than 80% (Fig. 6B; note how at a disease progression of 50%, the red line overlaps the black dotted line indicating near perfect calibration however at a recurrence of 80%, the red line and black dotted line do not overlap).

**Fig. 7:**
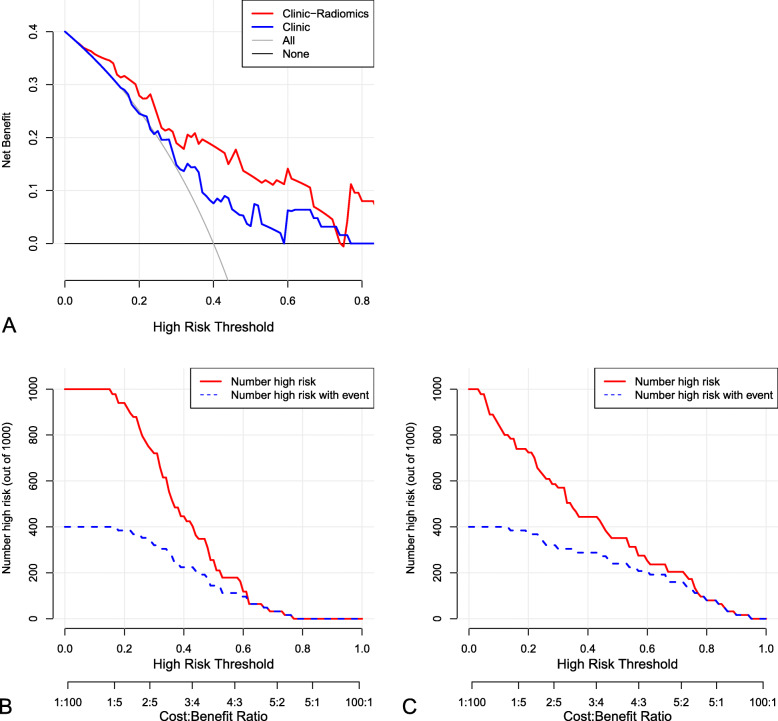
Decision curve analysis for Model L4 for the prediction of disease progression in patients with nasopharyngeal carcinoma (**A**). Clinical impact curves with variables in Model L4 and only 5 clinical variables in the training and validation cohorts (**B, C**)

The decision curves analysis (DCA) was used to estimate the clinical utility of Model L4, which integrated with 4 radiomic features and 5 clinical variables, based on the threshold probability. The threshold probability is used to derive the net benefit (defined as the fraction of true-positives subtracted by the fraction of false-positives weighted by the relative harm of a false-positive and false-negative result, Fig. 7). Graphical analysis of the net benefit against the threshold probability yields a decision analysis curve, which can then be used to assess the net benefit of nomogram-assisted decisions at different threshold probabilities, compared to the net benefit of decisions made with the assumption that either all or no patient has the outcome of interest (Fig. 7). When a patient’s threshold probability was within the range of 5 to 75%, the application of Model L4 added more net benefit than the “treat none” or “treat all” strategies, and also added net benefits of “clinical variables only”. Furthermore, we plotted clinical impact curve with variables in Model L4 and only 5 clinical variables separately (Fig. 7B and Fig. 7C). The results revealed that the predictive value of PDPM model was increased by incorporating both clinical variables and radiomic features.

## Discussion

In this study, we extracted radiomic features from pretreatment baseline MRI examinations, and constructed a machine learning model (PDPM model) by combining radiomic features and clinical variables to predict the disease progression of NPC patients after initial treatment. The PDPM Model L4 based on 4 radiomic and 5 clinical features showed good predictive performance, and its prognostic performance was significantly better than other models. We further presented and validated a radiomics nomogram that integrated variables in Model L4 for prediction of disease progression, which achieved satisfactory discrimination performance. The predictive value of PDPM model was increased by incorporating both clinical variables and radiomic features.

Several previous studies have made great efforts to evaluate the relationship between radiomics and clinical outcomes in NPC. Compared with previous studies of NPC on radiomics and disease progression in NPC, which focused only on risk of distant metastasis or local recurrence [5, 13], or was limited to NPC in T4 stage [12], our study included a wide range from early-stage NPC to locally advanced, but not distant metastatic NPC. Compared with the studies that only contoured the largest cross-section of the lesion [12], we obtained 3D VOIs in continuous multiple slices of the entire tumor, which may better contain the heterogeneity of the tumor. There is likely to be a discrepancy between radiomics features those obtained from the largest primary tumor cross-sectional area and the whole tumor. A study included 55 patients with primary colorectal cancer to determine whether there was a difference between contrast enhanced CT texture features from the largest cross-sectional area versus the whole tumor [26]. It demonstrated that whole tumor analysis may provide a more representative evaluation of tumor heterogeneity. The study of Yang also indicated that although 2D and 3D radiomics signature both had favorable prognosis, 3D signature had a better performance [27]. Although a clear superiority of 3D vs. 2D radiomics model performance remains to be scientifically proven, 3D analysis, by virtue of including the entire tumor volume, may better reflect overall tumor heterogeneity. The Dice similarity coefficient (DSC), which measures the degree of agreement between the three-dimensional consistency of tumor segmentation made by two or more readers [28], was calculated for absolute agreement between segmented VOIs in our study. In our study, patients were mainly from non-endemic areas of NPC, while in other studies [5, 29], cohort were mainly composed of homogeneous population from non-endemic regions. Moreover, our study adopted DCA to explain the best predictive model, making our results more favourable for clinical application.

We developed 8 models with two machine learning methods for predicting the 3-year disease progression of NPC. Based on the variable group 4, the Model L4 established by logistic regression algorithm could best discriminate patients who had developed disease progression. Interestingly, a significant difference was found in the prediction performance of Model L4 and Model L1. The 4 radiomic features and 5 clinical variables in Model L4 were selected from 13 radiomic features and 10 clinical variables by multivariate analysis. This result suggested that redundant features exist in a simple combination group [5].

The analysis identified “T2-w_wavelet-HLL_glszm_High Gray Level Zone Emphasis”, “CET1-w_wavelet-HLL_glszm_High Gray Level Zone Emphasis”, “CET1-w_wavelet-LHH_glszm_Gray Level Non-Uniformity”, and “CET1-w_wavelet-LHL_gldm_Large Dependence Low Gray Level Emphasis” as the most important factors of 13 radiomic features in predicting 3-year disease progression. The GLSZM analyzes the distance between groups of voxels with similar grey-levels by counting the number of groups of linked voxels [30]. The GLDM describes pair-wise arrangement of pixels with the set absolute difference in gray-level, in a given direction and distance, and used to highlight local heterogeneity information [31]. GLSZM and GLDM are regional texture features whose ability in differentiating patients with different prognosis has been already observed in various tumors [32, 33]. In line with those studies, the findings here suggest that the heterogeneity of intra-tumoral MRI distribution is an useful biomarker in predicting treatment outcome for patients with NPC. Besides, age, T stage, N stage, histology and smoking history were also found to be clear indicators of risk in the PDPM model, and these findings were consistent with conventional prior knowledge.

Features that were associated with disease progression would make a better and more interpretable model. To provide a clinically applicable method for individual prediction of 3-year disease progression, we further generated a radiomics nomogram that integrated 4 radiomic features and 5 clinical variables in PDPM Model L4. Our results showed that the radiomic nomogram provides better predictive performance compared with clinical nomogram (which only consisted of 5 clinical variables) and full features nomogram (which consisted of 4 radiomic features and 5 clinical variables). This finding was also supported by the results of the ROC analysis. The performance of the nomogram was further verified in the training cohort and validation cohort. The parameters of the nomogram can be easily acquired. Age, T stage, N stage, histology and smoking history were conventional predictive factors and could be obtained at the time of consultation. Moreover, the radiomic features could be obtained via common algorithms. In summary, our study showed that the nomogram can serve as either a scoring system or a visualization tool for disease progression prediction in patients with NPC, thus aiding physicians in rapid evaluation of the metastatic risk in the clinic.

There were some limitations to our study. First, this was a retrospective study with a relatively small sample and patients were included from a single-center. Due to our strict inclusion criteria, such patients with apparent artifacts on MR images were excluded from the study. The possible impact of the exclusion on the results was unknown, but this caused the limitations of the radiomics model established in our study in clinical application. A prospective study with large cohort will be needed to further confirm the conclusions of the current study. Second, it should be noted that we did not investigate the association between the plasma Epstein-Barr virus DNA copy number and disease progression in this study. Although this factor has been confirmed as a potentially useful predictor for the prognosis of NPC, this information was not available for most of our cohort. Third, in our study, we excluded areas of necrosis, hemorrhage, and cystic areas from VOIs, unlike some other studies of NPC. The impact of this practice on model performance and reproducibility might be an interesting topic for future research.

## Conclusions

In conclusion, we proposed a noninvasive radiomics model that incorporates the radiomics features and clinical variables to facilitate pretreatment prediction of disease progression in patients with NPC. Our radiomics model may facilitate clinical decision making and potentially improve survival outcomes in selected patients, which required further studies to explore the generalized utility of our model and translate it into clinical practice.

## Supplementary Information

**Additional file 1: Additional Figure 1.** An example of manual segmentation with exclusion for necrosis/cystic area (yellow arrow). A 45-year-old male patient with NPC. The segmented tumor is within the red contour in one slice of oblique axial T2WI/FS sequence (a) and the red contour in one slice of axial CE-T1WI sequence (b).

## Data Availability

The datasets used and/or analysed during the current study are available from the corresponding author on reasonable request.

## Abbreviations

AUC: area under the curve
LR: logistic regression
NPC: nasopharyngeal carcinoma
ROC: receiver operating characteristic
SVM: support vector machine
TNM: tumor-node-metastasis
