# Supplementary figures and images for: Baseline MRI-based radiomics model assisted predicting disease progression in nasopharyngeal carcinoma patients with complete response after treatment
Source: Cancer Imaging. 2022 Jan 28;22:10. doi: 10.1186/s40644-022-00448-4 (PMC8800208; doi:10.1186/s40644-022-00448-4)

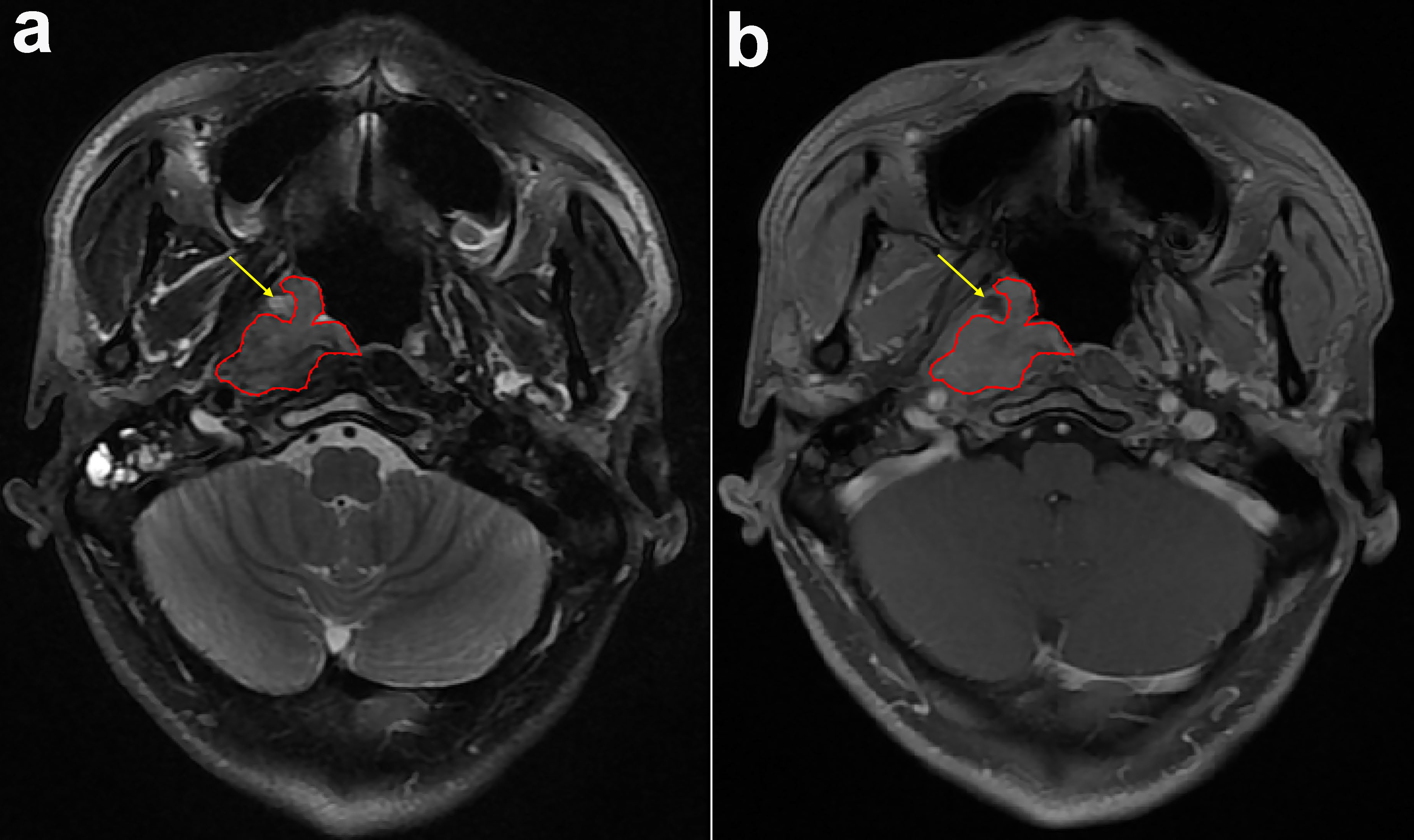

Supplement: Supplementary file 1 — Additional file 1: Additional Figure 1. An example of manual segmentation with exclusion for necrosis/cystic area (yellow arrow). A 45-year-old male patient with NPC. The segmented tumor is within the red contour in one slice of oblique axial T2WI/FS sequence (a) and the red contour in one slice of axial CE-T1WI sequence (b). [file 40644_2022_448_MOESM1_ESM.tif]
